# Draft Genome Sequence for *Desulfovibrio africanus* Strain PCS

**DOI:** 10.1128/genomeA.00144-13

**Published:** 2013-04-11

**Authors:** Steven D. Brown, Sagar M. Utturkar, Adam P. Arkin, Adam M. Deutschbauer, Dwayne A. Elias, Terry C. Hazen, Romy Chakraborty

**Affiliations:** Biosciences Division, Oak Ridge National Laboratory, Oak Ridge, Tennessee, USAa;; Graduate School of Genome Science and Technology, University of Tennessee, Knoxville, Tennessee, USAb;; Physical Biosciences Division, Lawrence Berkeley National Laboratory, Berkeley, California, USAc;; College of Engineering, University of Tennessee, Knoxville, Tennessee, USAd;; Earth Sciences Division, Lawrence Berkeley National Laboratory, Berkeley, California, USAe

## Abstract

*Desulfovibrio africanus* strain PCS is an anaerobic sulfate-reducing bacterium (SRB) isolated from sediment from Paleta Creek, San Diego, CA. Strain PCS is capable of reducing metals such as Fe(III) and Cr(VI), has a cell cycle, and is predicted to produce methylmercury. We present the *D. africanus* PCS genome sequence.

## GENOME ANNOUNCEMENT

Sulfate-reducing bacteria (SRB) are anaerobic microorganisms that play important roles in sulfur and carbon cycles in diverse environments (see reviews [1–3]). *Desulfovibrio africanus* strain PCS was isolated from a lactate/sulfate enrichment culture inoculated with sediment samples obtained from Paleta Creek, San Diego, CA. The isolated strain was Gram negative, motile, nonsporulating, and 99% similar by 16S rRNA gene sequencing to *Desulfovibrio africanus* subsp. *uniflagellum* (GenBank accession number EU659693) and *D. africanus* strain Walvis Bay (CP003221.1) (4), which is consistent with the species definition (5). *D. africanus* strains, including PCS, have different morphotypes associated with a cell cycle (6–10), and PCS incompletely oxidizes lactate, accumulating acetate as an end product.

*D. africanus* strains have been shown to methylate inorganic mercury [Hg(II)] to methylmercury (MeHg), a potent human neurotoxin (10–12). The capability to produce MeHg is found only in a subset of SRB and Fe(III)-reducing bacteria (IRB) (11–16). A 4.2-Mb complete genome sequence for *D. africanus* strain Walvis Bay (4, 17), which produces MeHg, has been reported (10, 12). Recently, genetic studies have shown that a two-gene cluster encoding a putative corrinoid-containing CO dehydrogenase/acetyl coenzyme A (acetyl-CoA) synthase, HgcA, and a 2[4Fe-4S] ferredoxin, HgcB, is required to produce MeHg in SRB and IRB (18). HgcA and HgcB are predicted to have roles as a methyl carrier and an electron donor, respectively. To date, strain PCS has not been tested for its ability to generate MeHg.

The genome sequence for strain PCS was generated using Illumina data, as described previously (19). Briefly, CLC Genomics Workbench (version 5.5) was used to trim 100-bp reads from a paired-end library for quality sequence data, and these were then assembled using Velvet (version 1.2.01) (20). The resulting assembly generated 45 DNA contigs for an estimated genome size of ~3.9 Mb. The maximum contig size was 609,036 bp, the average contig size was 87,322 bp, and the *N*_50_ was 140,584 bp. The average read depth was approximately 560× the estimated genome size. The draft genome sequence was annotated as previously described (21) and 3,561 candidate protein coding genes were predicted.

The PCS genome had a G+C content of 61.2%, which is similar to the 61.4% G+C content reported for strain Walvis Bay (4). Strain PCS shows 95% average nucleotide identity to strain Walvis Bay when the two genome sequences are compared using the JSpecies program (22). Strain PCS contains putative *hgcA* (PCS_01240) and *hgcB* (PCS_01242) genes that are ~97% and 98% identical, respectively, to their Walvis Bay counterparts at the nucleotide level. In both strains, a gene encoding a predicted radical *S*-adenosylmethionine (SAM) superfamily or Fe-S oxidoreductase protein is in a 3′ position relative to *hgcA* and 5′ relative to *hgcB*, a genetic organization that differs from those of other MeHg-producing bacteria like *D. desulfuricans* strain ND132 (16, 18). The *D. africanus* PCS genome sequence will facilitate further studies with this bacterium.

### Nucleotide sequence accession numbers. 

This whole-genome shotgun project has been deposited at DDBJ/EMBL/GenBank under the accession number AOSV00000000. The version described in this paper is the first version, AOSV01000000.

## References

[1] Keller KL Wall JD 2011 Genetics and molecular biology of the electron flow for sulfate respiration in *Desulfovibrio*. Front. Microbiol. 2:135 21747813 10.3389/fmicb.2011.00135 PMC3129016

[2] Muyzer G Stams AJM 2008 The ecology and biotechnology of sulphate-reducing bacteria. Nat. Rev. Microbiol. 6:441–454 18461075 10.1038/nrmicro1892

[3] Odom JM Peck HDJ 1984 Hydrogenase, electron-transfer proteins, and energy coupling in the sulfate-reducing bacteria *Desulfovibrio*. Annu. Rev. Microbiol. 38:551–592 6093686 10.1146/annurev.mi.38.100184.003003

[4] Brown SD Wall JD Kucken AM Gilmour CC Podar M Brandt CC Teshima H Detter JC Han CS Land ML Lucas S Han J Pennacchio L Nolan M Pitluck S Woyke T Goodwin L Palumbo AV Elias DA 2011 Genome sequence of the mercury-methylating and pleomorphic *Desulfovibrio africanus* strain Walvis Bay. J. Bacteriol. 193:4037–4038 21642452 10.1128/JB.05223-11 PMC3147520

[5] Campbell LL Kasprzycki MA Postgate JR 1966 Desulfovibrio africanus sp. n., a new dissimilatory sulfate-reducing bacterium. J. Bacteriol. 92:1122–1127 5927208 10.1128/jb.92.4.1122-1127.1966 PMC276386

[6] Chakraborty R Joyner D Wozei E Holman H-YN Lam S Hazen TC 2006 Desulfovibrio strain PCS, a novel metal reducing pleomorphic sulfate reducing bacterium, poster Q-166. Abstr. 106th Gen. Meet. Am. Soc. Microbiol. American Society for Microbiology, Washington, DC

[7] Chakraborty R Joyner DC Wozei E Holman HY Hazen TC 2006 *Desulfovibrio* strain PCS, a metal reducing pleomorphic sulfate reducing bacterium, abstr 1936. 11th Int. Symp. Microb. Ecol., Vienna, Austria, 20 to 25 August 2006.

[8] Chakraborty R Tang YJ Pingitore F Keasling JD Hazen T 2008 Metabolic pathways in the pleomorphic metal-reducing organism *Desulfovibrio africanus* strain PCS, poster K-054. Abstr. 108th Gen. Meet. Am. Soc. Microbiol. American Society for Microbiology, Washington, DC

[9] Jones HE 1971 A re-examination of *Desulfovibrio africanus*. Arch. Mikrobiol. 80:78–86 5126869 10.1007/BF00410582

[10] Moberly JG Miller CL Brown SD Biswas A Brandt CC Palumbo AV Elias DA 2012 Role of morphological growth state and gene expression in *Desulfovibrio africanus* strain Walvis Bay mercury methylation. Environ. Sci. Technol. 46:4926–4932 22500779 10.1021/es3000933

[11] Ekstrom EB Morel M FM Benoit JM 2003 Mercury methylation independent of the acetyl-coenzyme a pathway in sulfate-reducing bacteria. Appl. Environ. Microbiol. 69:5414–5422 12957930 10.1128/AEM.69.9.5414-5422.2003 PMC194973

[12] Gilmour CC Elias DA Kucken AM Brown SD Palumbo AV Schadt CW Wall JD 2011 The sulfate-reducing bacterium *Desulfovibrio desulfuricans* ND132 as a model for understanding bacterial mercury methylation. Appl. Environ. Microbiol. 77:3938–3951 21515733 10.1128/AEM.02993-10 PMC3131654

[13] Fleming EJ Mack EE Green PG Nelson DC 2006 Mercury methylation from unexpected sources: molybdate-inhibited freshwater sediments and an iron-reducing bacterium. Appl. Environ. Microbiol. 72:457–464 16391078 10.1128/AEM.72.1.457-464.2006 PMC1352261

[14] Compeau GC Bartha R 1985 Sulfate-reducing bacteria: principal methylaors of mercury in anoxic estuarine sediment. Appl. Environ. Microbiol. 50:498–502 16346866 10.1128/aem.50.2.498-502.1985 PMC238649

[15] King JK Kostka JE Frischer ME Saunders FM 2000 Sulfate-reducing bacteria methylate mercury at variable rates in pure culture and in marine sediments. Appl. Environ. Microbiol. 66:2430–2437 10831421 10.1128/aem.66.6.2430-2437.2000 PMC110551

[16] Brown SD Gilmour CC Kucken AM Wall JD Elias DA Brandt CC Podar M Chertkov O Held B Bruce DC Detter JC Tapia R Han CS Goodwin LA Cheng J-F Pitluck S Woyke T Mikhailova N Ivanova NN Han J Lucas S Lapidus AL Land ML Hauser LJ Palumbo AV 2011 Genome sequence of the mercury-methylating strain *Desulfovibrio desulfuricans* ND132. J. Bacteriol. 193:2078–2079 21357488 10.1128/JB.00170-11 PMC3133056

[17] Hurt RA Brown SD Podar M Palumbo AV Elias DA 2012 Sequencing intractable DNA to close microbial genomes. PLoS One 7:e41295 doi:10.1371/journal.pone.0041295 22859974 10.1371/journal.pone.0041295 PMC3409199

[18] Parks JM Johs A Podar M Bridou R Hurt RA Smith JD Tomanicek SJ Qian Y Brown SD Brandt CC Palumbo AV Smith JC Wall JD Elias DA Liang L 2013 The genetic basis for bacterial mercury methylation. Science 339:1332–1335 23393089 10.1126/science.1230667

[19] Brown SD Utturkar SM Klingeman DM Johnson CM Martin SL Land ML Lu TYS Tse-Yuan S Schadt CW Doktycz MJ Pelletier DA 2012 Twenty-one genome sequences from *Pseudomonas* species and 19 genome sequences from diverse bacteria isolated from the rhizosphere and endosphere of *Populus deltoides*. J. Bacteriol. 194:5991–5993 23045501 10.1128/JB.01243-12 PMC3486089

[20] Zerbino DR Birney E 2008 Velvet: algorithms for de novo short read assembly using de Bruijn graphs. Genome Res. 18:821–829 18349386 10.1101/gr.074492.107 PMC2336801

[21] Markowitz VM Chen I-MA Palaniappan K Chu K Szeto E Grechkin Y Ratner A Jacob B Huang J Williams P Huntemann M Anderson I Mavromatis K Ivanova NN Kyrpides NC 2012 IMG: the integrated microbial genomes database and comparative analysis system. Nucleic Acids Res. 40:D115–D122 22194640 10.1093/nar/gkr1044 PMC3245086

[22] Richter M Rosselló-Móra R 2009 Shifting the genomic gold standard for the prokaryotic species definition. Proc. Natl. Acad. Sci. U. S. A. 106:19126–19131 19855009 10.1073/pnas.0906412106 PMC2776425

